# Intra-seasonal variation in feeding rates and diel foraging behaviour in a seasonally fasting mammal, the humpback whale

**DOI:** 10.1098/rsos.211674

**Published:** 2022-07-06

**Authors:** Ross C. Nichols, David E. Cade, Shirel Kahane-Rapport, Jeremy Goldbogen, Alison Stimpert, Douglas Nowacek, Andrew J. Read, David W. Johnston, Ari Friedlaender

**Affiliations:** ^1^ Institute of Marine Sciences, Long Marine Laboratory, University of California, Santa Cruz. 115 McAllister Way, Santa Cruz, CA 95060, USA; ^2^ Department of Biology, Hopkins Marine Station, Stanford University, 120 Ocean View Boulevard, Pacific Grove, CA 93950, USA; ^3^ Moss Landing Marine Laboratories, San Jose State University, 8272 Moss Landing Road, Moss Landing, CA 95039, USA; ^4^ Nicholas School of the Environment and Earth Sciences & Pratt School of Engineering, Duke University Marine Laboratory, 135 Duke Marine Lab Road, Beaufort, NC 28516, USA; ^5^ Nicholas School of the Environment and Earth Sciences, Duke University Marine Laboratory, 135 Duke Marine Lab Road, Beaufort, NC 28516, USA

**Keywords:** humpback whale, foraging ecology, seasonal foraging, fasting mammal, Antarctic, biologging

## Abstract

Antarctic humpback whales forage in summer, coincident with the seasonal abundance of their primary prey, the Antarctic krill. During the feeding season, humpback whales accumulate energy stores sufficient to fuel their fasting period lasting over six months. Previous animal movement modelling work (using area-restricted search as a proxy) suggests a hyperphagic period late in the feeding season, similar in timing to some terrestrial fasting mammals. However, no direct measures of seasonal foraging behaviour existed to corroborate this hypothesis. We attached high-resolution, motion-sensing biologging tags to 69 humpback whales along the Western Antarctic Peninsula throughout the feeding season from January to June to determine how foraging effort changes throughout the season. Our results did not support existing hypotheses: we found a significant reduction in foraging presence and feeding rates from the beginning to the end of the feeding season. During the early summer period, feeding occurred during all hours at high rates. As the season progressed, foraging occurred mostly at night and at lower rates. We provide novel information on seasonal changes in foraging of humpback whales and suggest that these animals, contrary to nearly all other animals that seasonally fast, exhibit high feeding rates soon after exiting the fasting period

## 1. Introduction

In seasonally variable systems, resources are unevenly distributed throughout the year, resulting in periods of resource surplus and deficit [1]. During a surplus, seasonally fasting mammals accumulate excess energy as internal (e.g. fat, blubber) or external (e.g. food hoarding) energy caches [2]. Animals can then use these energy reserves when resources in the environment are insufficient to maintain homeostasis or fuel energetically costly life-history events (e.g. hibernation, gestation, migration, moult, arousal) [3,4]. Seasonally fasting animals often undergo a period of hyperphagia, concentrating food intake over a relatively short period of time, resulting in a peak rate of mass gain during the foraging season [2,4]. Timing of the hyperphagic period is critical in understanding how seasonally fasting mammals develop lipid stores necessary for growth and survival. Collectively, these concepts embody what we introduce as the fasting animal framework: a collection of ecological models previously developed for fasting animals that we apply as a comparative ecological tool.

Humpback whales (*Megaptera novaeangliae)*, are one of the largest seasonally fasting mammals, and the most well-studied whale species, but little work has focused on the fasting animal framework. Humpback whales partition the year between low-latitude breeding and calving sites in winter and early spring and high-latitude foraging sites between late spring and autumn. Migrations between these sites can span thousands of kilometres [5]. Humpback whales are thought to largely fast during the breeding season and during migration, with all metabolic needs during that time being satisfied by the breakdown of internal adipose stores (mostly in blubber). Humpback whales are estimated to lose 25–50% of their body mass over this period [6]. Unlike terrestrial fasting animals that reduce energy expenditure through torpor, humpback whales and other migratory baleen whales continue to incur normal, and perhaps increased, metabolic costs while fasting by engaging in costly physiological and behavioural processes associated with reproduction and migration [7]. While many aspects of humpback whale behaviour probably aim to minimize energetic costs during the fasting period [8], the cost compared with fasting mammals that engage in torpor is relatively high.

Humpback whales feed via a specialized feeding apparatus that enables the engulfment of prey-laden water, the volume of which is nearly equivalent to the animal's body mass in a single mouthful. The ability for bulk consumption of small-bodied prey enables highly efficient foraging of dense prey patches [9]. Southern Hemisphere foraging humpback whales exploit dense aggregations of Antarctic krill (*Euphausia superba*) in highly productive Antarctic and sub-Antarctic waters as annual sea ice retreats. Humpback whale distributions mirror geographical variation in the seasonal distribution of Antarctic krill [10–12]. However, seasonal variation in the vertical distribution of krill remains poorly understood due, in part, to a lack of longitudinal seasonal data. All future reference to seasons in the Southern Hemisphere is that of their astronomical definitions: summer [January–March]; autumn [April–June]; winter [July–September]; spring [October–December]. Available evidence suggests krill biomass in continental shelf waters is concentrated deeper in autumn compared with spring and summer. Additionally, in fall, krill appear to perform diel vertical migrations (DVM) over larger vertical distances and are present at the surface for less hours of the day than in spring and summer [13–15].

To date, research on the seasonality of Antarctic humpback whale feeding behaviour has been limited to low-resolution measurements using state-space animal movement models based on satellite-derived locations. Under this approach, animal movement is classified into behavioural states, such as ‘travelling’ or ‘area restricted search’ (ARS). ARS, associated with low movement speed and high turn angles of the animal, is commonly assumed to be an indicator of foraging, as animals are remaining in the same area for extended periods of time. However, ARS also includes non-foraging behaviours that present similar movement characteristics (e.g. resting, social) and cannot distinguish individual foraging events. Weinstein and Friedlaender [16] used temporal changes in ARS behaviour of humpback whales in the waters around the Western Antarctic Peninsula (WAP) to suggest a continuous increase in time spent foraging from summer to autumn, with the greatest amount of time spent in ARS occurring at the end of the foraging season. ARS of humpback whales foraging off East Antarctica, however, found peak ARS occurring in February–March [17], earlier in the feeding season. If foraging inferred from time spent in ARS is assumed to be a reasonable proxy of food intake, it can be hypothesized that humpback whale food intake similarly should increase throughout the foraging season [16], or peak in mid-feeding season [17]. Conversely, early studies estimating humpback whale weight gain from industrial whaling records predicted that body mass would be at a seasonal asymptote by February (early/mid-season). Contradicting the inference from ARS estimates, this would require peak food intake in January or earlier [6]. Resolution of these inconsistencies requires continuous, accurate measures of feeding rates throughout the course of the entire feeding season. To date, however, the only published literature on fine-scale foraging behaviour of these whales in the WAP comes from the autumn (May–June), when whales exhibit extreme diel foraging patterns, restricting foraging to night, mostly at depths greater than 50 m [18,19].

Humpback whales provide a unique case within the fasting animal framework. Among the seasonally fasting mammals, humpback whales incur one of the highest absolute energetic costs during the fasting period [3]. However, when and how this energy is accumulated over their foraging season is virtually unknown. Additionally, because they target high-density prey and are able to locate such aggregations throughout the water column (e.g. Friedlaender *et al*. [18,20]) baleen whale foraging can aid in investigating the seasonal variation of prey presence/behaviour in the water column. Using high-resolution motion-sensing tags, we investigated humpback whale foraging dynamics over the feeding season to test the hypothesis that humpback whale food intake (inferred from ARS) increases as the feeding season progresses from January to June. To investigate these questions, we analysed hourly tag data using a hurdle model approach, by fitting generalized additive mixed models (GAMM) for both foraging presence/absence and feeding rates separately. Additionally, we pooled data temporally into discrete analysis periods for visualization and investigation of broad trends in intra-seasonal humpback whale feeding behaviour.

## 2. Material and methods

We deployed two types of high-resolution, archival, non-invasive, digital recording motion-sensing tags to study the foraging behaviour of humpback whales: (i) DTAGs (http://www.animaltags.org/)—which record depth (pressure transducer), and motion (triaxial magnetometry and accelerometry) sampled at 50 Hz, and audio (stereo-hydrophone; sampling rate: 48–64 kHz) [21]; and (ii) CATS (Customized Animal Tracking Solutions; http://www.cats.is/)—which record depth (pressure transducer), motion (triaxial magnetometry and accelerometry) sampled between 40 and 400 Hz. Select tags recorded audio (stereo hydrophone; sampling rate: 64 kHz) and/or video [22].

**Figure 1.**
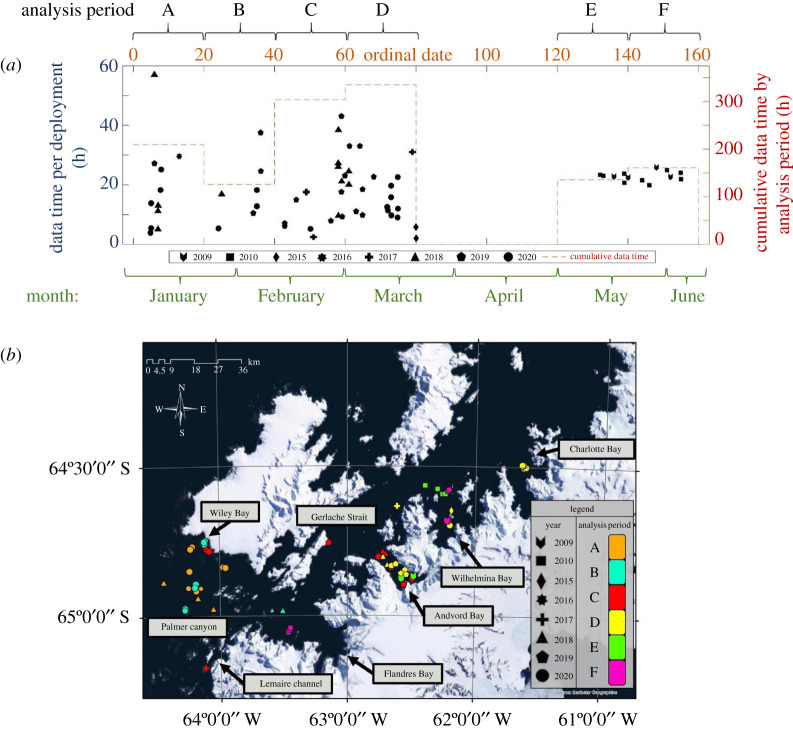
Study period and site. (*a*) Each point represents a tag deployment (left *y*-axis). Red dashed lines are cumulative values (right *y*-axis). Analysis periods A–F, encompass 20-day calendar periods: (A) 1–20 January, (B) 21 January–9 February, (C) 10 February–1 March, (D) 2–21 March, (Excluded; 22 March–30 April), (E) 1–20 May, (F) 21 May–9 June. (*b*) Study site and tag deployments (plotted points) by year and analysis period.

We deployed 83 tags between 5 January and 4 June during the years 2009, 2010 and 2016–2020. Sixteen deployments were DTAGs deployed in 2009 and 2010; the remaining 67 were CATS tags deployed between 2016 and 2020. All tags were deployed on non-calf humpback whales in nearshore waters on the continental shelf of the Western Antarctic Peninsula (WAP) between 64°32′7.74″ S and 65°6′44.83″ S latitude and 61°29′2.70″ W and 64°50′47.12″ W longitude (figure 1). Data processing methodologies were consistent across years and tag types. Tags were deployed from 6–7 m inflatable vessels, typically by a scientist in a bow pulpit using a 3–4 m carbon fibre pole with a friction-fit housing. Tags were attached to the surface of whales with four silicone suction cups. DTAGs were programmed to release after 24 h post deployment. CATS tags used a 24 h dissolvable release or detached from the whale from suction loss up to 57 h later. GPS locations were taken immediately after deployment. After detaching from the animal and floating to the surface, a GPS location and VHF transmitters were used to relocate the tag for retrieval and data download.

### 2.1. Data processing and lunge detection

Post-recovery, raw data were extracted using proprietary software developed by the respective tag companies. Sensor data processing for use in further analysis used custom Matlab (Natick, MA: The MathWorks Inc., v. 2014a) scripts [21,22]. Individual feeding lunges were manually identified through a combination of accelerometer, magnetometer and speed data. Animal speed, used to identify lunges, was calculated from accelerometer jiggle or flow noise [23], depending on the sample rate of the accelerometer. Expert auditors, experienced in identifying characteristic kinematic signals of foraging, manually discriminated lunges in each deployment. Idiosyncratic signals of lunges included episodic fluctuations in body orientation (roll, pitch and heading), peaks in jerk (m s^−3^) and speed (m s^−1^). Fourteen deployments with less than one full hour of data or with malfunctioning sensors were excluded from subsequent analysis.

### 2.2. Analysis periods

To examine broad seasonal and diel trends in feeding rates and foraging behaviour, data were pooled into 20-day analysis periods to estimate foraging metrics characteristic of each binned period. We chose 20-day bin durations to balance deployment sample size with seasonal resolution. We performed our analyses on various other bin sizes; however, no significant differences in general trends were found except when deployment sample sizes became extremely low (less than three).

We created six 20-day analysis periods (figure 1*a* and table 1) from 1 January to 9 June (ordinal 1–160). The period from 22 March to 30 April (ordinal 81–120) was excluded due to insufficient data. Feeding rates were calculated as lunges per unit time and were calculated for every hour and half-hour of tag data. We pooled all individual hourly feeding rates and lunge depths by analysis period and calculated mean hourly feeding rates and mean feeding depths for each hour of the day (24 bins, 0000–2300).

**Table 1.** Foraging metric statistics by analysis period. Estimated daily feeding rates are reported as number of lunges day^−1^ whale^−1^. Mean hourly feeding rates are reported as number of lunges hour^−1^ whale^−1^; **‘**hourly N’ represents the range of hourly samples for each hour of the day by analysis period displayed as mean (min–max).

	analysis period					
	A	B	C	D	E	F
ordinal date range	1–20	21–40	41–60	61–80	121–140	141–160
number of deployments	11	7	17	20	6	7
tag data hours	217.6	169.2	274.8	382.4	105.0	102.9
hourly N	7.3 (5–10)	5.1 (3–7)	11.5 (7–15)	13.0 (9–19)	5.8 (4–6)	6.8 (5–7)
mean hourly feeding rate	24.7	25.5	29.5	25.7	15.5	26.0
N	264	168	408	480	144	168
s.d.	21.1	25.9	30.6	26.9	20.1	28.2
range (min – max)	0–86.0	0–108.0	0–113.0	0–116.9	0–60.9	0–108.0
mean feeding depth (m)	16.1	12.1	55.4	101.0	96.3	116.5
N	6989	3862	9242	7999	2153	2759
s.d.	17.4	8.9	63.1	107.8	65.6	91.5
range (min – max)	0–157.3	0–111.8	0–301.1	0–461.1	3.0–339.5	1.4–387.7

### 2.3. The hurdle model

To investigate seasonal effects and relationships persistent throughout the feeding season, we employed a two-part hurdle model approach. The hurdle method assumes that the presence/absence of foraging and rate of feeding are controlled by different mechanisms and, thus, were modelled separately [24]. We then conceptually integrated the two model outputs and applied them to our research questions. Part I of our hurdle model included hourly tag data of foraging presence and absence as a response variable with a binomial distribution. Part II included non-zero hourly feeding rates from tag data as a response variable with a Gaussian distribution. The two-part method proved advantageous for our data, as attempts at fitting an integrated model were rejected upon attempting to control for assumptions of independence. These violations were alleviated upon applying the hurdle model approach.

For both parts of the hurdle model, we used the *mgcv* package [25] in R (v. 4.1.3, www.cran.r-project.org) to run the GAMMs. In both model parts, all covariates used (table 2) were applied as smoothers using thin plate regression splines, except for HOD (hour of the day) which used a cyclic spline. We ran correlation matrices to determine collinearity. Covariates with high collinearity (*r* > 0.7) were removed, and the covariate with greater biological relevance and interest was selected to remain in the model. Models were optimized using a backwards removal approach, removing the least significant terms (significance threshold, *p* < 0.05) and using the Akaike information criterion (AIC) as a determinant for removal of the covariate. Our dataset included multiple observations from individual deployments, therefore assumptions of independence had to be accounted for. To do this, we incorporated WID (an identification code unique to each deployment) as a random intercept to account for individual variability in both models. Additionally, to address independence between hourly observations within deployments, we applied an AR1 temporal autocorrelation structure using HSD (number of hours since the beginning of the respective deployment) nested by WID. Model assumptions including homogeneity, normality and independence were validated by plotting standardized residuals versus fitted values and versus each covariate in the model; we assessed the residuals for temporal and spatial dependency.

**Table 2.** GAMM covariates: covariates used in the two-part hurdle model investigating the foraging presence and feeding rates of Antarctic humpback whales. MLD was not included in the foraging presence/absence modelling.

covariate	abbreviation
ordinal date of the observation	DOY
hour of observation in local time (UTC – 3)	HOD
mean solar elevation during observation hour (°)	MSE
centroid solar elevation during observation hour (°)	CSE
year of deployment	YEAR
latitude of deployment (dd)	LAT
longitude of deployment (dd)	LON
mean depth of foraging lunges (m)	MLD
time since beginning of deployment (h)	HSD
deployment identification code	WID

All figures and statistical analyses were generated using statistical software Matlab, Esri ArcMap 9 (v. 10.8.1) and R package ‘Tidyverse’ [26]. Solar elevations were calculated using ‘The Climate Data Toolbox for MatLab’ [27] at the position of tag deployments.

## 3. Results

Of the 69 deployments (electronic supplementary material, appendix S1: table S2) included in analysis a total of 1276.4 h of recorded sensor data were usable. The length of data while on the animal ranged from 2.1 to 56.9 h with a mean (mean ± s.d.) duration of 18.49 ± 10.39 h. A total of 33 246 lunge feeding events were detected across all deployments. Lunges were detected at depths between 0 and 461 m (62.0 ± 81.28 m).

### 3.1. Intra-seasonal variation in feeding rate and depth

**Figure 2.**
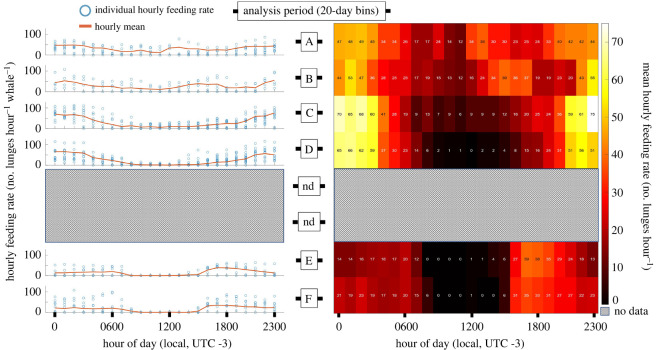
Seasonal feeding rates by hour of the day. The left panel shows plots for each analysis period (vertically stacked) with hourly feeding rates (*y*-axis) by hour of the day (*x*-axis) for every hour of sampled tag data. Individual hourly feeding rates are represented as a blue circle. The 40-day period between analysis period D and E is labelled as ‘nd’ for ‘no data’.

The analysis period approach found that maximum mean hourly feeding rates (lunges hour^−1^ whale^−1^) occurred in period C, 29.5 ± 30.6 (0–113.0) with minimum mean hourly feeding rates occurring in period E, 15.5 ± 20.1 (0–64.0) (table 1). Extremely low mean hourly feeding rates (less than 5 lunges hour^−1^ whale^−1^) occurred during select hours of analysis periods D (0900–1500), E (0800–1400) and F (0900–1400) (figure 2). No foraging was detected during select hours of period E (0800, 0900, 1100) and F (1000, 1100, 1300, 1400).

**Figure 3.**
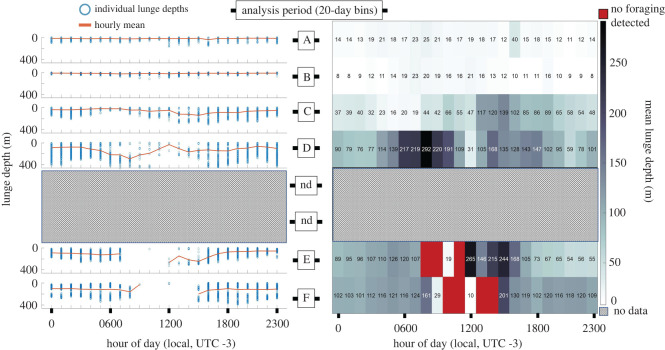
Seasonal feeding depths by hour of the day. The left panel shows plots for each analysis period (vertically stacked) with lunge depth (*y*-axis) by hour of the day (*x*-axis). Individual lunge depths are represented as a blue circle. The 40-day period between analysis period D and E is labelled as ‘nd’ for ‘no data’.

Maximum mean foraging depths occurred in period F at 116.4 ± 91.5 m (1.4–387.8) with minimum mean feeding depths occurring in period B with 12.1 ± 8.9 m (0–111.8) (figure 3 and table 1). Unimodal near surface (less than 30 m) foraging occurred in the beginning of the foraging season but shifted to a bimodal distribution with predominantly deeper lunges (greater than 50 m) later in the foraging season (electronic supplementary material, appendix S1: figure S1).

Feeding occurred during all hours at shallow depths in the summer before becoming progressively deeper and at lower rates as the season progressed into autumn; except for the hours surrounding midnight, which saw a peak in feeding rates in the late-summer/early autumn (figures 2 and 3).

### 3.2. The hurdle model

For both models, YEAR (year of deployment) and CSE (centroid solar elevation, i.e. solar elevation at 30-minute mark of each hour) covariates were removed, prior to fitting, due to correlation coefficients over the threshold with DOY (ordinal date of deployment) and MSE (mean solar elevation), respectively. Therefore, the total covariates (table 2) included in both model parts were DOY, HOD, MSE, LAT (deployment latitude) and LON (deployment longitude). MLD (mean lunge depth) was included in part II only.

**Figure 4.**
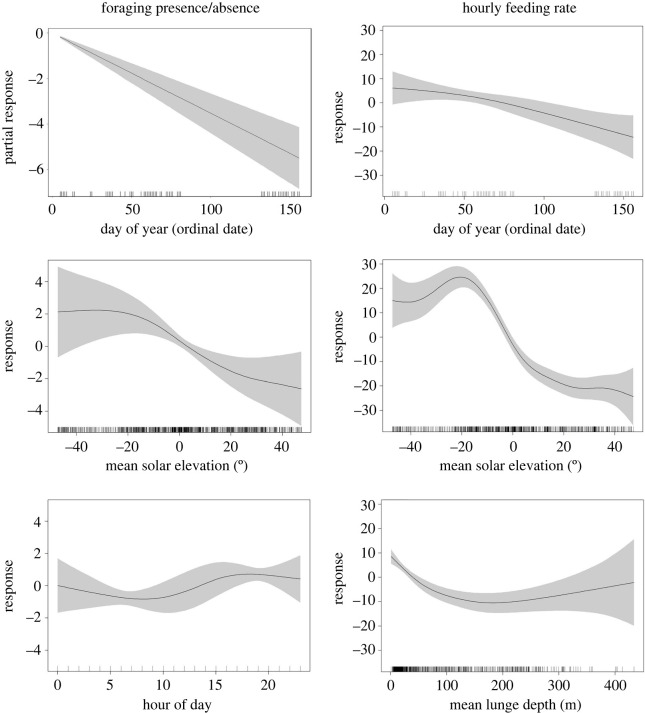
GAMM Response Plots for Humpback Whale Foraging: The left column shows response curves for the three covariates included in the foraging presence/absence GAMM. The right column shows response curves for the three covariates included in the hourly feeding rate GAMM. Shaded areas represent 95% confidence intervals. Rug plots on the *x*-axis of each plot show individual observations.

Part I—presence/absence of foraging: the results of the first model (*R*^2^ = 0.263, *n* = 1225) found two significant smoothing covariates (table 2), MSE and HOD. DOY was significant, but the model indicated a negative linear relationship and DOY was ultimately included as a parametric covariate. Two non-significant covariates (LAT, LON) were removed from the model. The AR1 correlation structure was kept, as the temporal autocorrelation was relatively large (*φ* = 0.53) and its inclusion lowered AIC considerably. The results of the model (figure 4) suggest that the probability of foraging is greater during hours with low light (MSE; empirical distribution function (edf) = 4.01, *F* = 5.07, *p* < 0.05), with an extreme shift in foraging probability centred at 0° MSE, which can be interpreted as sunset/sunrise. This indicates that foraging was most likely to occur at night, or between sunset and sunrise throughout the foraging season. Additionally, a significant effect was found for hour of day (HOD; edf = 2.98, *F* = 2.89, *p* < 0.05), but high confidence intervals relative to the response make interpretation of HOD difficult. However, some effect of HOD is present, especially when comparing between morning and afternoon hours. A significant, linear, partial effect was found for ordinal date (DOY; estimate = −0.035, s.e. = 0.009, *t* = −4.05, *p* < 0.05), indicating a negative seasonal effect on the presence of foraging. In other words, the probability for foraging to occur during any hour peaks at the beginning of the feeding season, progressively declining to the end of the feeding season.

Part II – hourly feeding rate: the results of the second model (*R*^2^ = 0.337, *n* = 870) included three significant smoothing covariates, DOY, MSE and MLD. Three non-significant covariates (LAT, LON, HOD) were ultimately removed from the model. The AR1 correlation structure was kept, as the temporal autocorrelation was relatively large (*φ* = 0.62) and including it improved AIC. The results of the model (figure 4) suggest that ordinal date (DOY; edf = 1.60, *F* = 7.75, *p* < 0.05) has a significant effect on hourly feeding rates over the foraging season. The seasonal effect we see in DOY suggests that hourly feeding rates become lower from the beginning to the end of the foraging season, with a strong decline occurring after ordinal date 65 (approx. 6 March). Additionally, hourly feeding rates were much higher in lower light conditions (MSE; edf = 6.85, *F* = 31.13, *p* < 0.05), with an extreme decline in the response centred around −5° solar elevation. The MSE response indicates that high hourly feeding rates were most closely associated with night-time, while lower feeding rates occurred in the daytime. Our model results also found that hourly feeding rates were highest at shallower average foraging depths (MLD; edf = 3.83, *F* = 10.92, *p* < 0.05). Feeding rates declined with increasing depth until appearing to asymptote at approximately 100 m, while high confidence intervals make the relationship at deeper depths unclear.

While no issues occurred in model validation for either model, we believe the addition of unavailable covariates related to specific prey distributions (e.g. patch depth/dimensions) and availability (e.g. patch presence/density) would probably improve the model overall. See electronic supplementary material, appendix S1: table S3 for extended model output statistics.

## 4. Discussion

Here we provide a fine-scale evaluation of seasonal variation in foraging behaviour of humpback whales in the Southern Ocean. In early summer, humpback whales exhibited extremely high feeding rates on shallow prey patches. As the foraging season progressed, whales subsequently fed less often, at lower rates and at deeper depths. Whales fed during all hours in the early foraging season (January), but only between 1800 and 0700 in the late season (June). During hours of low light, significantly higher feeding rates/presence were detected throughout the foraging season. These effects would seemingly favour foraging in the late feeding season (autumn) when there are more low-light hours. However, this benefit is offset by deeper feeding and increasing day of year, which greatly reduce feeding presence and rates from the beginning to the end of the foraging season. We posit that these changes in foraging behaviour are largely in response to changing prey availability (shallow versus deep) and the energetic needs of whales returning from a long period of fasting and acquiring energy as fast as possible. However, intrinsic factors such as satiation and foraging motivation may also play a role in seasonal shifts of foraging behaviour.

### 4.1. In relation to the fasting animal framework

Our data suggest that the most important period of humpback whale foraging effort occurs in early summer, soon after arriving in Antarctica. The high likelihood of foraging presence paired with high feeding rates during this period were probably facilitated by the continuous availability of shallow prey. In terrestrial systems, fasting animals typically express a hyperphagic period near the temporal midpoint between emergence and immergence from torpor [2,4]. This is the case in golden-mantled ground squirrels (*Callospermophilus lateralis*) and yellow-bellied marmots (*Marmota flaviventris*) [28]. The hyperphagic period can also occur near the end of the foraging season, as in some bear species [29]. The timing of the hyperphagic period in terrestrial mammals has been attributed to (i) the necessary increasing of metabolism after emergence from torpor results in muted behaviour for a period after emergence, also called ‘walking hibernation’ in bears, which limits activity and forging motivation [30], and (ii) the trade-off between the ability to accumulate sufficient energy caches before immergence into torpor with the detrimental costs of carrying those caches. Carrying internal adipose tissues incurs an increased cost of transport, limits mobility, increases predation risk and makes heat dissipation more difficult [1]. Thus, many terrestrial animals will optimize the acquisition of energy stores by modulating food intake to minimize the time spent carrying them while maximizing the energy accumulated before undergoing torpor. Humpback whales are largely unconstrained by these limitations due to a fully aquatic life and a likely absence of metabolic suppression [31], a low cost of transport [32,33] and a reduced likelihood of adult predation [34]. Therefore, it is advantageous for humpback whales to regain energy stores as soon as possible by maximizing foraging effort when prey is most efficient to consume.

We present a novel ecological perspective on how fasting animals manage the extreme nature of this life-history strategy. Research on humpback whale foraging has, thus far, focused on extrinsic controls. However, study of intrinsic controls of foraging behaviour is less common, in part because endogenous measures on free-living whales are difficult. By incorporating humpback whales into a fasting animal framework, we offer a comparative method of study on intrinsic influences of foraging behaviour. Evidence suggests that intrinsic controls govern fasting animal foraging behaviour on seasonal time scales [2,35]. Specifically, foraging motivation is managed via circannual rhythms, an endogenous ‘biological clock’ for annual life cycles that are attuned to the timing of events such as the hyperphagic period [2,36]. The hyperphagic period occurs when the intrinsic motivation to forage maximizes overlap with the extrinsic availability of prey, resulting in peak annual food intake. It is currently unknown if humpback whales exhibit circannual rhythms. Current methodologies of testing circannual rhythms require the animal to be captive and thus are untenable in humpback whales [36]. Rather, we test for the presence and timing of a hyperphagic period, a strong indication of intrinsic influence on seasonal foraging behaviours.

The decline in feeding rates we report over the foraging season is a product of both intrinsic and extrinsic influences; we posit that peak feeding rates in the early season indicate high intrinsic foraging motivation and support an early hyperphagic hypothesis. Future research would benefit from testing this through better measures of food intake, baseline measures of endogenous chemical signals related to foraging (e.g. free fatty acids, leptin, insulin, ghrelin) and measurements of humpback whale foraging prior to our study period, in the late spring (December), when the earliest whales begin to arrive to the feeding grounds. Our results also imply that disruption or disturbance to the early feeding season could have a disproportionate effect on a critical period of energy store development. When considering current trends of increasing anthropogenic presence in the Antarctic system [37], we recommend future work examines disturbance on an intra-seasonal scale to best assess potential impact.

### 4.2. Intra-seasonal variation in humpback whale foraging behaviour

Our results contrast with previous satellite telemetry studies [16,17]; we found that actual feeding exhibits the opposite trend, declining significantly and continuously as the foraging season progressed. While the satellite tag data does detect a shift in the distribution of foraging whales, the changes in ARS do not appear to reflect true feeding rates but rather other behavioural states including resting which could be interpreted as ARS. In the early season, the targeting of shallow, but horizontally dispersed prey could attenuate the ARS signal as humpback whales forage over broader areas [11]. In the late foraging season, krill concentrate coastally along the WAP and whales follow them into these embayments [11,12]. Accordingly, low movement behaviours (e.g. resting) become common during the daylight hours while deep foraging occurs solely at night. The combination of daytime resting and deep foraging in concentrated areas at night probably contributes to the increased amount of time whales spend in ARS but the relatively low feeding rates observed during this time of year.

Our results are not the first fine-scale measures that challenge the assumption of ARS-inferred foraging. In wandering albatross (*Diomedea exulans*), ARS was associated more with environmental conditions than with actual prey captures [38], and in southern elephant seals (*Mirounga leonina*), foraging success was more closely related to areas of historical prey availability than ARS [39]. Our findings emphasize the call for additional fine-scale behavioural measures to complement and validate more coarse measures of foraging behaviour such as ARS [40]. Our results, however, do not aid in resolving key differences in reported ARS of humpback whales between the WAP [16] and the East Antarctic [17]. Differing environmental conditions (embayments versus ice edge; respectively) and population-level differences (e.g. migration pathways, demographics) [41] are worthy of future comparative investigation. Additionally, recent observations along the WAP showed seasonal variation in the demographics of humpback whales in this region [42], consistent with previous studies [43], as well as a high proportion of annually breeding females [42]. Together, these temporal and demographic shifts are probably driven by specific energetic requirements [6], which could influence seasonal foraging behaviours and strategies. Thus, the relationship between an individual's demography and foraging behaviour deserves further investigation.

### 4.3. Foraging behaviour and its relation to the prey environment

**Figure 5.**
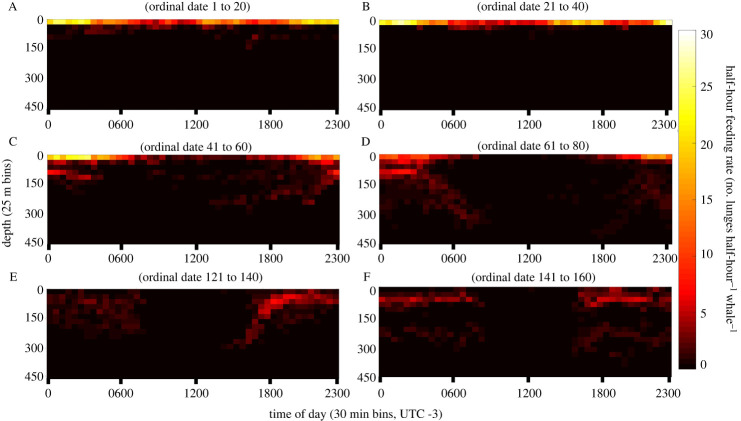
Heatmap of feeding rates by depth and time of day for each analysis period. Heatmaps of half-hour feeding rates over the diel period. Each plot presents data across 20-day analysis periods (A–F). Figures for ordinal dates 81–120 are absent due to insufficient data.

We believe that seasonal fluctuations in prey availability near the surface are likely drivers of shifts in humpback whale foraging behaviour [14,44]. Cresswell *et al*. [14] found that, in the summer months along the WAP, krill are largely distributed in surface waters (less than 60 m) throughout the night and day, with relatively higher abundance of shallow krill occurring at night. These observations are similar to our reporting of humpback whale feeding (figures 3 and 5) and also correspond to reporting of seasonal shifts [13], and temporally discrete reports of krill depths over the foraging season [15,45]. However, prey abundance must be accompanied with a minimum threshold patch density at the scale of whale mouthfuls [46] for foraging to be energetically beneficial for baleen whales [47]. Thus, additional information on vertical distributions of prey on diel and seasonal time scales are needed to assess this as a driver of humpback whale foraging behaviour.

While foraging at depth, baleen whale feeding rates are reduced due to increased travel times to/from the surface and the inability to overlap filtration and breathing cycles [19,47]. However, our feeding rate model indicates that the depth of prey alone does not fully account for the decline in feeding rates observed. Our model found significant responses in both feeding rate and feeding presence to mean solar elevation and suggests a component of diel variability in foraging behaviour persists throughout the entire study period. However, the magnitude of differences in diel foraging behaviour appears more pronounced later in the season, with very little foraging (less than or equal to 6 lunges hour^−1^ whale^−1^) occurring between 08.00 and 15.00 in analysis periods D–F. While the proximate cause of reduced feeding rates during the midday hours in the late season is uncertain, the reduced presence and lack of detected feeding during 30% of the day signals the general abatement of feeding behaviour as the foraging season progresses.

## 5. Conclusion

Our results challenge previous hypotheses of late and mid-season humpback whale hyperphagia by demonstrating that the presence of foraging and feeding rates peak in early summer and decline in autumn. Early in the summer, humpback whales forage during all hours of the day at shallow depths, becoming deeper during the day and into autumn. How these seasonal changes in feeding rates affect food intake is uncertain but highlights the potential importance of the early feeding season in seasonal energy acquisition. An early hyperphagic period is unique among fasting mammals and is probably enabled by the availability of high-quality prey, high foraging motivation and minimal constraints on carrying internal energy caches.

## Data Availability

Data used in analyses and novel code used for this manuscript are available at doi:10.6084/m9.figshare.15062619. Electronic supplementary material is available online [48].
